# Loss of renal olfactory receptor 1393 leads to improved glucose homeostasis in a type 1 diabetic mouse model

**DOI:** 10.14814/phy2.15007

**Published:** 2021-12-08

**Authors:** Alexis R. Schiazza, Elizabeth G. Considine, Madison Betcher, Blythe D. Shepard

**Affiliations:** ^1^ Department of Human Science Georgetown University Washington District of Columbia USA

**Keywords:** diabetes, glucose reabsorption, olfactory receptor, sex, SGLT1, SGLT2

## Abstract

Renal olfactory receptor 1393 (Olfr1393) is an understudied sensory receptor that contributes to glucose handling in the proximal tubule. Our previous studies have indicated that this receptor may serve as a regulator of the sodium glucose co‐transporters (SGLTs) and contributes to the development of glucose intolerance and hyperfiltration in the setting of diet‐induced obesity. We hypothesized that Olfr1393 may have a similar function in Type 1 Diabetes. Using Olfr1393 wildtype (WT) and knockout (KO) mice along with streptozotocin (STZ) to induce pancreatic β‐cell depletion, we tracked the development and progression of diabetes over 12 weeks. Here we report that diabetic male Olfr1393 KO mice have a significant improvement in hyperglycemia and glucose tolerance, despite remaining susceptible to STZ. We also confirm that Olfr1393 localizes to the renal proximal tubule, and have uncovered additional expression within the glomerulus. Collectively, these data indicate that loss of renal Olfr1393 affords protection from STZ‐induced type 1 diabetes and may be a general regulator of glucose handling in both health and disease.

## Graphical abstract

We previously found that olfactory receptor 1393 is expressed in the renal proximal tubule where it contributes to glucose homeostasis. Here we show that loss of this receptor offers protection from the development of type 1 diabetes. This study adds to the growing appreciation for the roles that sensory receptors play in seemingly non‐sensory tissues.

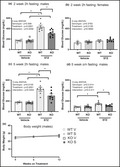

## 1 INTRODUCTION

Diabetes is an endocrine‐based disease approaching epidemic proportions. Although this disease is multifactorial, this chronic condition is characterized by the development of hyperglycemia due, in part, to the insufficient production of (Type 1 Diabetes; T1D) or response to (Type 2 Diabetes; T2D) insulin (Gale & Gillespie, 2001; Thomas et al., 2016). The increase in circulating blood glucose levels gives rise to a multitude of co‐morbidities including increased blood pressure, heightened risk of cardiovascular disease, and most prominently, renal failure (Forbes & Cooper, 2013).

The kidney has emerged as a target for the control of blood glucose in patients with both T1D and T2D. Under euglycemic conditions, blood glucose is freely filtered at the glomerulus and completely reabsorbed along the lumen of the proximal tubule by two sodium‐glucose co‐transporters, SGLT1 and SGLT2 (Mather & Pollock, 2010, 2011; Shepard & Pluznick, 2017). Glycosuria can occur when blood glucose levels rise above the handling capacity of the proximal tubule, and inhibitors that target SGLT2, or both SGLT1 and SGLT2, take advantage of this by promoting glycosuria as a novel method for improving hyperglycemia (Marsenic, 2009; Mather & Pollock, 2010; McCrimmon & Henry, 2018; Shepard & Pluznick, 2017). Along with the improved glucose tolerance, patients with diabetes have found these inhibitors to be beneficial in reducing the risk of cardiovascular events and limiting diabetes‐induced hyperfiltration which has been linked to the progression of diabetic kidney disease (Vallon et al., 2013, 2014; Wanner et al., 2016).

We recently identified a G protein‐coupled receptor (GPCR), Olfactory Receptor 1393 (Olfr1393), that is expressed in the renal proximal tubule where it contributes to glucose homeostasis (Shepard et al., 2016). Olfr1393 knockout (KO) mice were found to have mild glycosuria and improved glucose tolerance along with a reduction in luminal expression of SGLT1 in the proximal tubule. When these mice were challenged with a high fat diet to induce early stages of diet‐induced obesity, the male KOs had improved glucose tolerance, an attenuation in glomerular hyperfiltration, and decreased expression of SGLT2 as compared to their wildtype (WT) littermates (Shepard et al., 2019). Collectively, these previous studies have established Olfr1393 as a regulator of renal glucose handling and a contributing factor in the development of T2D.

Given our previous findings, we reasoned that Olfr1393 KO mice may be similarly protected from the development of T1D. To test this, Olfr1393 WT and KO mice were challenged with Streptozotocin (STZ) to induce pancreatic β‐cell depletion and were monitored for the development and progression of diabetes. Indeed, we found that while Olfr1393 KO mice remained sensitive to STZ, hyperglycemia was significantly attenuated and they had improved glucose tolerance. Collectively, our data further establish Olfr1393 as a critical player in the diabetic arena.

## 2 MATERIALS AND METHODS

### 2.1 Olfr1393 mouse model and streptozotocin treatment

Olfr1393 WT and KO mice (both males and females) were generated as described previously on a C57BL6 background (Shepard et al., 2016). Mice were maintained on a 12:12‐h light‐dark cycle, with normal chow and water provided ad libitum. All studies performed on the WT and KO mice were age‐ and littermate‐matched and included both males and females with all analysis performed by sex. To induce T1D, 12‐week‐old male and female mice were fasted overnight (16 h) and randomly assigned STZ or the vehicle control. Working stocks of STZ were made fresh with ice cold, sterile 1× PBS immediately before use at a concentration of 10 mg/ml. Mice were weighed daily and injected intraperitoneally with 55 mg STZ/kg body weight for five consecutive days. Vehicle controls were administered an equal volume of 1× PBS in parallel. Two weeks following the last injection of STZ, mice were fasted for 2 h (~8–10 am) and their blood glucose levels were determined using a glucometer. Only those mice that reached a 2 h fasting blood glucose value of >230 mg/dl were deemed diabetic and were included in the rest of the study. Our overall diabetic success rate was >90%.

### 2.2 Glucose and insulin tolerance tests

At 2, 5, and 12 weeks following STZ injections, mice were subjected to glucose and insulin tolerance tests (GTT and ITT). For the GTTs, mice were fasted overnight for 16 h (at the 2‐ and 5‐week timepoints; ~5 pm to 9 am) or for 6 hours (for the 12‐week timepoint; ~8 AM to 2 PM) and injected with glucose (1 g/kg body weight IP; Sigma). For the ITTs, mice were fasted for 2 h (~8–10 am) and were injected with recombinant human insulin (0.7 U/kg body weight IP; ThermoFisher). To track tolerance, blood was collected from a tail nick and glucose values were measured with a glucometer (Accu‐Chek Guide; Roche) at 0, 15, 30, 60, 90, and 120 min post‐injection. The 2 h fasting blood glucose values reported in the results were obtained from the time 0 of the ITT while the overnight fasting blood glucose values were obtained from time 0 of the overnight fasted GTT.

### 2.3 Glomerular filtration rate

Glomerular filtration rate (GFR) was measured in conscious, unrestrained mice by transcutaneous measurement of FITC‐sinistrin (MediBeacon, Mannheim, Germany) prior to induction of diabetes (week 0) and at 5 and 12 weeks post‐STZ as previously described (Schreiber et al., 2012; Shepard et al., 2016, 2019). All GFR measurements were performed between 8 am and 12 pm. Briefly, at least 24 h before the procedure, fur on a small area on the back of the mouse was shaved and completely removed using a depilatory (Nair). On the day of the measurement, the transcutaneous measurement device was attached to the hairless region of the back and a baseline (>1 min) measurement was obtained; the mice were then retro‐orbitally injected with 7.5 mg/kg body weight FITC‐Sinistrin (MediBeacon), returned to their cage, and had free access to food and water for 1 h. Thereafter, the measuring device was removed, and data were downloaded and analyzed using the three‐compartment model (Friedemann et al., 2016). GFR was analyzed both as μl/min/100 g BW and by μl/min by calculating the rate constant for each individual mouse as previously described (Schreiber et al., 2012; Shepard et al., 2019).

### 2.4 Histology analysis

At the conclusion of the study (12‐weeks post‐STZ challenge), spot urine was collected and analyzed by dipstick analysis (Roche) to detect changes in urinary glucose excretion as a consequence to hyperglycemia induced by STZ. The mice were then euthanized via CO_2_ asphyxiation and both the kidneys and pancreas were removed, drop‐fixed in 10% buffered formalin for 24 h, embedded in paraffin, and processed for Periodic Acid Schiff (PAS; kidneys) or Hematoxylin and Eosin (H&E; pancreas) staining by the Histopathology Shared Resources at Georgetown University Lombardi Comprehensive Cancer Center. For glomerular injury, stained kidneys were imaged (EVOS; ThermoFisher) at both 10× and 40× with five fields of view imaged/kidney. Glomerular size was determined using ImageJ software analysis. Briefly, each glomerulus on the 40× images was encircled and the area (both with and without the Bowman's Space) was measured. To quantify mesangial expansion, 40× PAS images were deconvoluted in ImageJ. The threshold of PAS staining was set, converted to 8‐bit grayscale, and mean intensity was calculated for each individual glomeruli. To quantify pancreatic islet area, H&E stained pancreases were imaged at 20× with islet area measured by ImageJ. Islet area was normalized to total pancreatic area to account for differences in size of the tissue collected. Tissues were harvested from ~2 pm to 4 pm.

### 2.5 TUNEL staining

Paraffin‐embedded pancreas sections were deparaffinized with Xylenes and re‐hydrated with decreasing concentrations of ethanol washes (90% ethanol, 80% ethanol, 50% ethanol, 35% ethanol). Slides were treated with 20 mg/ml proteinase K for 30 min, permeabilized with 0.1% Triton‐X 100 for 2 min at 4℃, and stained with the TUNEL reagent (Sigma) at 37℃ for 1 hour according to manufacturer's protocol. Stained slides were washed 3× with 1× PBS and mounted with vectashield. Images were taken at both 10× and 20× (EVOS; ThermoFisher) and mean fluorescence intensity for each islet was measured using ImageJ.

### 2.6 BaseScope

At the conclusion of the study, kidneys from Olfr1393 WT and KO diabetic and vehicle control mice were obtained following perfusion fixation with 100 mL phosphate buffered 4% paraformaldehyde (PFA) solution and post‐fixed in 4% PFA for 24 h at 4°. Samples were then equilibrated to a 10% sucrose solution, followed by a 20% sucrose solution, followed by a 30% sucrose solution for at least 6 h or until tissue sank to the bottom of tubes, embedded in paraffin, and sectioned (14 μm) by the Histopathology Shared Resources at Georgetown University Lombardi Comprehensive Cancer Center. Prior to performing BaseScope, slides were deparaffinized using xylenes, followed by dehydration using a series of ethanol washes. Olfr1393 mRNA was localized by BaseScope using a custom‐made probe and the manufacturer's recommended protocol (Advanced Cell Diagnostics). Briefly, following pretreatment and a 10‐min hydrogen peroxide treatment, target retrieval was performed for 15 min at 100℃, followed by protease treatment for 30 min at 40℃ (Protease IV solution of BaseScope kit; Advanced Cell Diagnostics). Probes (Olfr1393: BA‐MM‐Olfr1393‐EJ; positive control: BA‐MM‐Ppib‐122) were then hybridized for 2 h at 40℃ followed by amplification steps 1–8 using the BaseScope Detection Reagents. FastRed detection was applied as per manufacturer instructions along with 50% Gill's Hematoxylin I counterstain for 2 min to visualize nuclei. Sections were then briefly transferred to 0.02% ammonia water, washed with tap water, dried at 60℃ for 15 min and mounted using VectaMount mounting medium (Vector Laboratories). Positive and negative control probes were used in this study with 1‐ZZ Hs‐POLR2A acting as a positive control, and 1‐ZZ DapB acting as a negative control. Images were captured within one week of experimental procedure using an Invitrogen EVOS LF Auto Imaging system at 40× magnification.

### 2.7 C‐peptide

Mice were fasted for 6 h (~9 am to 3 pm) prior to receiving an intraperitoneal injection of glucose (1 g/kg BW). 15 min after challenge, blood was collected via a submandibular facial vein in heparin‐coated tubes. Plasma was isolated via centrifugation (2000 ×*g* for 15 min at 4℃) and 5 μl was used to perform c‐peptide ELISA according to manufacturer's protocol (Crystal Chem). Data were plotted as calculated (in ng/ml).

### 2.8 Western blotting

Whole kidney was lysed in 10% w/v isolation solution as previously reported (Ecelbarger et al., 2000). Following clearance at 16,000 ×*g* for 15 min at 4℃, gel samples were placed in Laemmli sample buffer. 40 μg protein was loaded/sample, resolved by gel electrophoresis, and transferred to PVDF. To probe for KIM‐1 (also known as T cell‐immunoglobulin‐mucin; R&D Systems, Minneapolis, MN AF1817), NGAL (Neutrophil Gelatinase‐Associated Lipocalin; R&D Systems AF1757), and β‐actin (Abcam), samples were blocked in 5% nonfat dried milk containing 0.1% Tween‐20 (blotto) for 30 min, incubated overnight with appropriate antibodies in blotto (1:2000), and detected using corresponding secondary antibodies (ThermoFisher). Chemiluminescence was developed (General Electric Lifestyle) and visualized (BioRad). Intensity was normalized to β‐actin expression. Kidney lysates obtained from a kidney injury model using 2,8‐dihydroxyadenine (Klinkhammer et al., 2020) was used as a positive control for the presence of KIM‐1 and NGAL. To probe for SGLT1 and SGLT2, samples were blocked in 5% nonfat dried milk containing 0.01% Tween‐20 for 30 min, incubated overnight with rabbit‐SGLT1 or rabbit‐SGLT2, both at 1:1000; (Balen et al., 2008; Vallon et al., 2011) in 5% BSA, and detected using corresponding secondary antibodies (ThermoFisher). Chemiluminescence was developed (General Electric Lifestyle) and visualized (BioRad). Total expression of SGLT1 and SGLT2 was determined using densitometry analysis (ImageJ) and normalized to β‐actin expression.

### 2.9 Statistics

Values presented are means ± standard error. Two‐way ANOVA followed by Sidak's post‐hoc test was performed when multiple comparisons were required (i.e. GFR for genotype and different time points, blood glucose values) significance was set at *p* < 0.05. A three‐way ANOVA with time noted as a repeated variable was performed for the ITT and GTTs. For the 12‐week GTT (which began following a 6 h fast), some of the blood glucose values exceeded the limit of detection of the glucometer. For this analysis, the maxed‐out values were omitted and a mixed method analysis was performed. A one‐way ANOVA was performed when comparing values between WT V, WT STZ, KO V, and KO STZ groups. All statistical tests were performed using Prism GraphPad.

## 3 RESULTS

**FIGURE 1:**
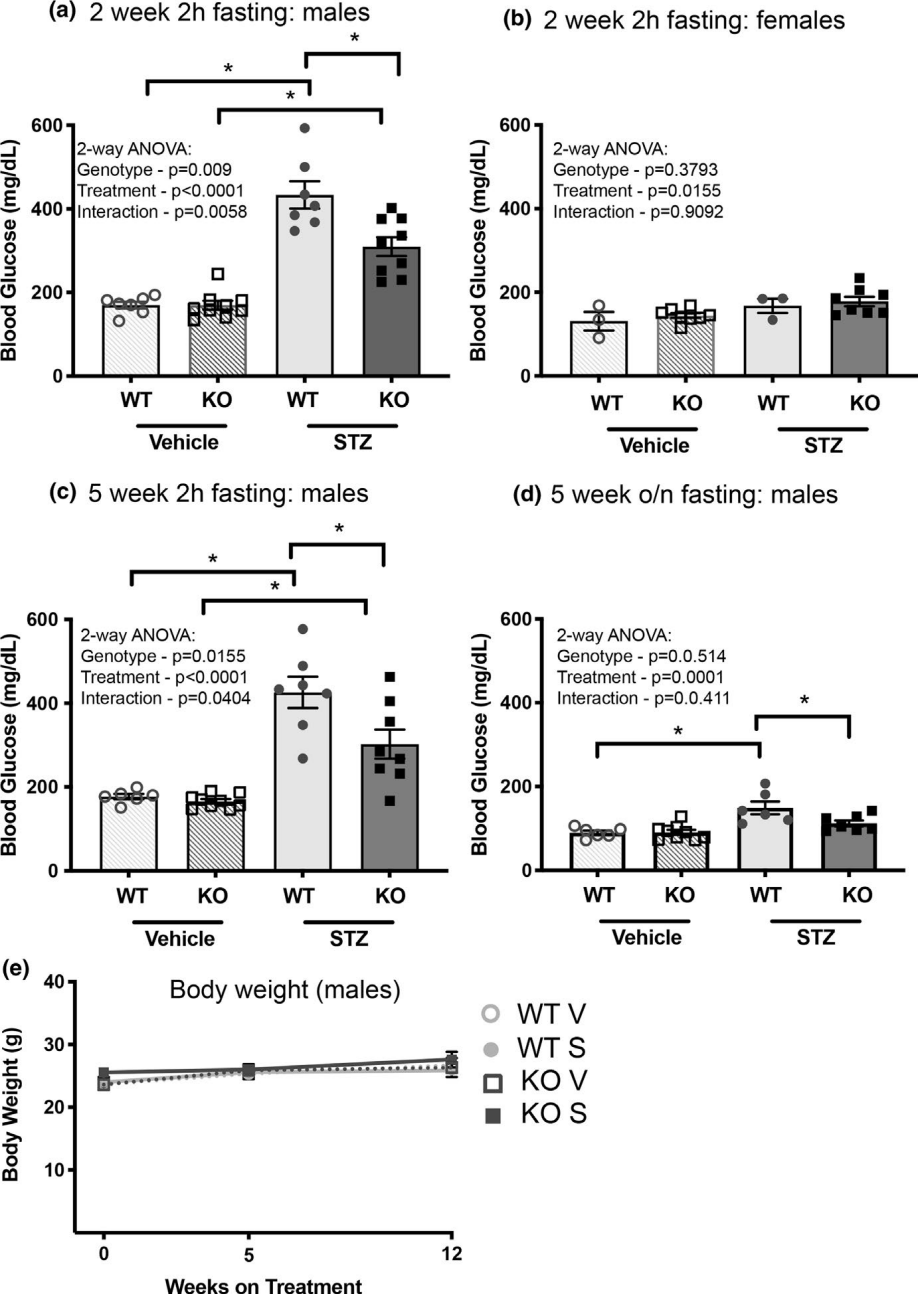
Diabetic Olfr1393 KO mice present with attenuated hyperglycemia. Olfr1393 WT and KO mice were challenged with Streptozotocin (STZ) or vehicle to induce T1D. 2 weeks following challenge, male WT mice displayed significant hyperglycemia after a 2 hour fast which was attenuated in the diabetic KO mice (vehicle: 6 WT, 9 KO; STZ: 7 WT, 9 KO) (a). The STZ‐injected female mice did not display signs of developing T1D (vehicle: 3 WT, 8 KO; STZ: 3 WT, 8 KO) (b). Attenuated hyperglycemia remained evident in the males 5 weeks post‐STZ (vehicle: 6 WT, 9 KO; STZ: 7 WT, 8 KO) (c) and was accompanied by a significant reduction in overnight fasting blood glucose levels (vehicle: 6 WT, 9 KO; STZ: 6 WT, 7 KO) (d). *p < 0.05 by two‐way ANOVA followed by Sidak's multiple comparisons test. Despite an attenuation in hyperglycemia, there were no changes observed in body weight (e). Values represent mean ± SEM

To induce T1D in Olfr1393 WT and KO mice, 12‐week‐old male and female mice were challenged with low‐dose injections of STZ (55 mg/kg BW) or vehicle for 5 consecutive days. Animals that presented with a 2‐hour fasting blood glucose of >230 mg/dl two‐weeks post‐STZ challenge were deemed diabetic and continued with the study. While male Olfr1393 WT and KO mice developed robust hyperglycemia as compared to their vehicle controls, the female mice were unaffected by STZ treatment (Figure 1a,b). This was characterized by a lack of hyperglycemia (Figure 1b) and normal glucose tolerance at both 2 and 5 weeks post‐STZ. Thus, only the diabetic male mice were included in further analysis.

To follow the development of T1D in our male Olfr1393 WT and KO mice, we monitored fasting blood glucose at both 2‐ and 5‐weeks post‐injection. Two weeks following STZ‐challenge, both WT and KOs exhibited hyperglycemia compared to the vehicle controls although none of the mice reached the threshold requiring insulin dosing. However, this was significantly attenuated in the KOs (Figure 1a). This phenotype persisted at 5 weeks (Figure 1c) where even the 16 h overnight fasting blood glucose levels were notably lowered (Figure 1d). Collectively, these data indicate that loss of Olfr1393 can promote improved blood glucose control in the setting of T1D. Despite this, there were no significant differences in weight gain between diabetic WT and KO mice up to 12 weeks post‐STZ, possibly due to the use of a low‐dose regimen (Figure 1e).

**FIGURE 2:**
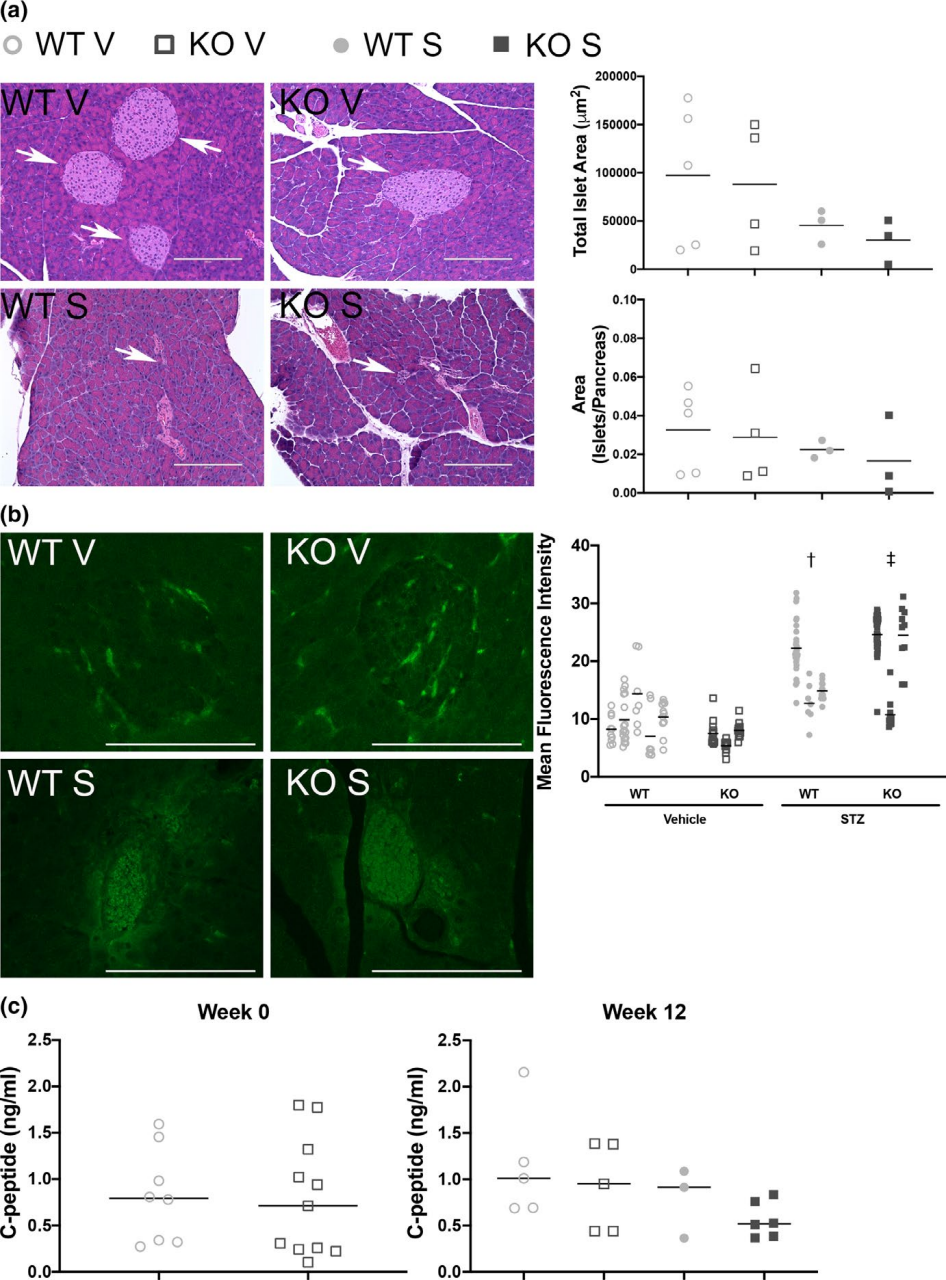
Olfr1393 KO mice are susceptible to STZ‐challenge. 12 weeks after STZ challenge, the pancreas was removed from both WT and KO vehicle (V) and STZ‐treated (S) mice. H&E histological analysis was performed and islets (arrows) were visualized (a). Total islet area (in μm^2^; top right) was plotted and normalized to total pancreas area (bottom right). No differences between STZ‐treated Olfr1393 WT and KO mice were detected (vehicle: 5 WT, 4 KO; STZ: 3 WT, 3 KO). TUNEL staining was performed on the same pancreas samples to detect apoptotic islets (b). Both WT and KO‐STZ challenged mice showed an increase in the mean fluorescence intensity of their islets (†*p* < 0.05 WT vehicle vs WT STZ; ‡*p *< 0.05 KO vehicle vs KO STZ by one‐way ANOVA followed by Tukey's post‐hoc test). Scale bar = 100 μm (vehicle: 5 WT, 3 KO; STZ: 3 WT, 3 KO). Circulating c‐peptide levels were measured following a glucose challenge (c) at either week 0 (prior to STZ injections; *N* = WT: 8, KO: 11) or week 12 (*N* = vehicle: 5 WT, 5 KO; STZ: 3 WT, 6 KO). In all graphs, the median is marked by a solid horizontal line

To rule out the possibility that Olfr1393 KO mice are resistant to STZ challenge, histological analysis of the pancreatic islets was completed at the conclusion of the study (Figure 2a). Total area of the observed islets was calculated; as expected, this was reduced in the STZ‐treated mice although there were no differences between the diabetic WT and KO pancreata (Figure 2a top right). To account for any differences in the total pancreatic area quantified, we also calculated the ratio of islet area/total pancreas area (Figure 2a bottom right). No statistical differences were identified suggesting that Olfr1393 KO mice are equally susceptible to STZ challenge. To confirm that STZ challenge promoted islet cell apoptosis, TUNEL staining was performed; islets from both STZ‐treated WT and KO mice exhibited increased apoptosis as indicated by an increase in the mean fluorescence intensity of TUNEL‐positive nuclei (Figure 2b). Measured insulin levels were too low to be quantified; thus, we sought to measure the release of c‐peptide following a glucose challenge. Prior to STZ injection (week 0), there were no differences in circulating c‐peptide levels between Olfr1393 WT and KO mice (Figure 2c). This is in keeping with earlier reports that circulating insulin levels are not altered in these mice (Shepard et al., 2016). At 12‐weeks post‐STZ challenge, there was a trend toward decreased c‐peptide release in STZ‐treated mice, but no differences noted between Olfr1393 WT and KO mice.

**FIGURE 3:**
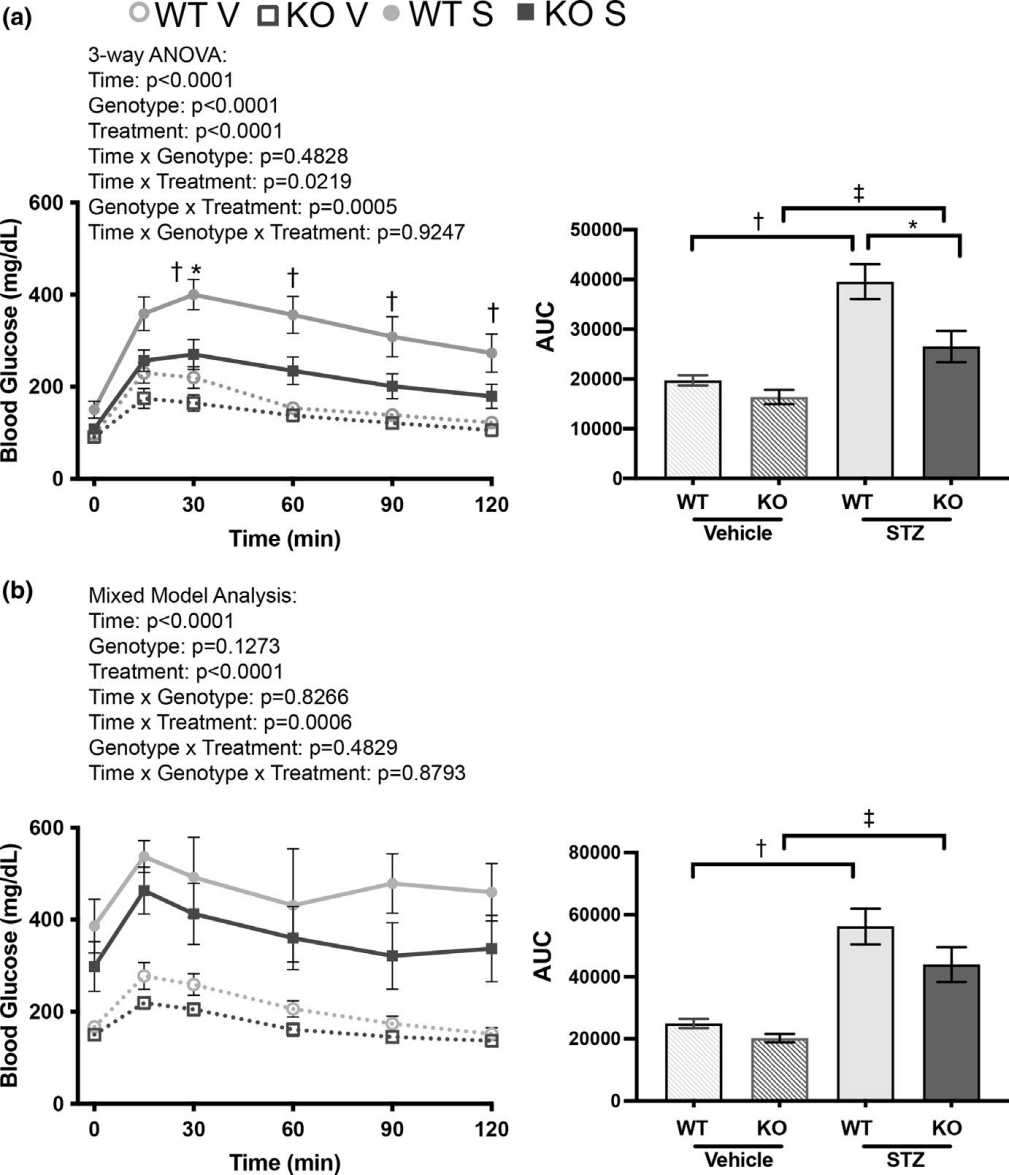
Glucose tolerance is improved in male diabetic Olfr1393 KO mice. 5 weeks after STZ challenge, Olfr1393 WT and KO mice were subjected to a glucose tolerance test (GTT) following a 16 h overnight fast. While both diabetic WT and KO mice displayed significant glucose intolerance compared to vehicle controls, this was significantly improved in the diabetic KOs (Three‐way ANOVA followed by Tukey's post‐hoc test: *p<0.05 WT STZ vs KO STZ; †*p* < 0.05 WT vehicle vs WT STZ; ‡*p* < 0.05 KO vehicle vs KO STZ). The improvement is also observed in the area under the curve on the righthand column with significance defined as *p < 0.05 WT STZ vs KO STZ; †*p* < 0.05 WT vehicle vs WT STZ; ‡*p* < 0.05 KO vehicle vs KO STZ by one‐way ANOVA followed by Tukey's post‐hoc test (vehicle: 4 WT, 9 KO; STZ: 5 WT, 8 KO) (a). 12 weeks after STZ challenge, mice were subjected to a GTT following a more physiological fast of 6 h. Values that exceeded the limit of detection on the glucometer were omitted; mixed method analysis and area under the curve indicate significant impaired glucose tolerance in STZ‐challenged mice (†*p* < 0.05 WT vehicle vs WT STZ; ‡*p* < 0.05 KO vehicle vs KO STZ by one‐way ANOVA followed by Tukey's post‐hoc test). Vehicle: 4 WT, 7 KO; STZ: 5 WT, 6 KO (b)

Olfr1393 KO mice on both a normal chow and high fat diet (Shepard et al., 2016, 2019) exhibit an improved glucose tolerance. Given the attenuation in hyperglycemia in our T1D mice, we sought to determine if this tracked with a change in glucose tolerance. Thus, WT and KO mice were subjected to a glucose tolerance test (GTT) at 5 and 12 weeks post‐STZ. By 5 weeks, the diabetic WT and KO mice were severely glucose intolerant as compared to their vehicle controls; nonetheless, the diabetic KO mice outperformed WT males in clearing a glucose challenge following an overnight fast (Figure 3a). Compared to the diabetic WT mice, the KOs had a significantly lower peak glucose value and returned to or near baseline at a faster rate. This improvement was also observed by calculating the area under the curve (AUC) for the diabetic mice (Figure 3a). Studies have shown differences in glucose tolerance following mild, moderate, and severe fasting states with overnight fasting characterized by an increase release of stress hormones inducing a pathophysiological condition (Jensen et al., 2013). Thus, to determine if the improved glucose tolerance was also observed following a more physiological fast, we performed a GTT after a 6‐h fast on the mice 12‐weeks post‐STZ. Under these conditions, a marked difference between the vehicle and diabetic mice was observed; both the diabetic cohorts were severely glucose intolerant characterized by significant hyperglycemia and the inability to clear the glucose bolus 2 h after the test began (Figure 3b). The glucometer used for this study has a maximum measurable glucose value of 600 mg/dl, and given the severity of the hyperglycemia, this value was routinely met. In fact, the majority (3/5) of the WT diabetic mice had their peak glucose values above 600 mg/dl while this was a rarer occurrence for the KO diabetics (2/6). However, given the limit of the glucometer, we were unable to determine if there was an improvement between the WT and KO diabetics at this later diabetic timepoint.

**FIGURE 4:**
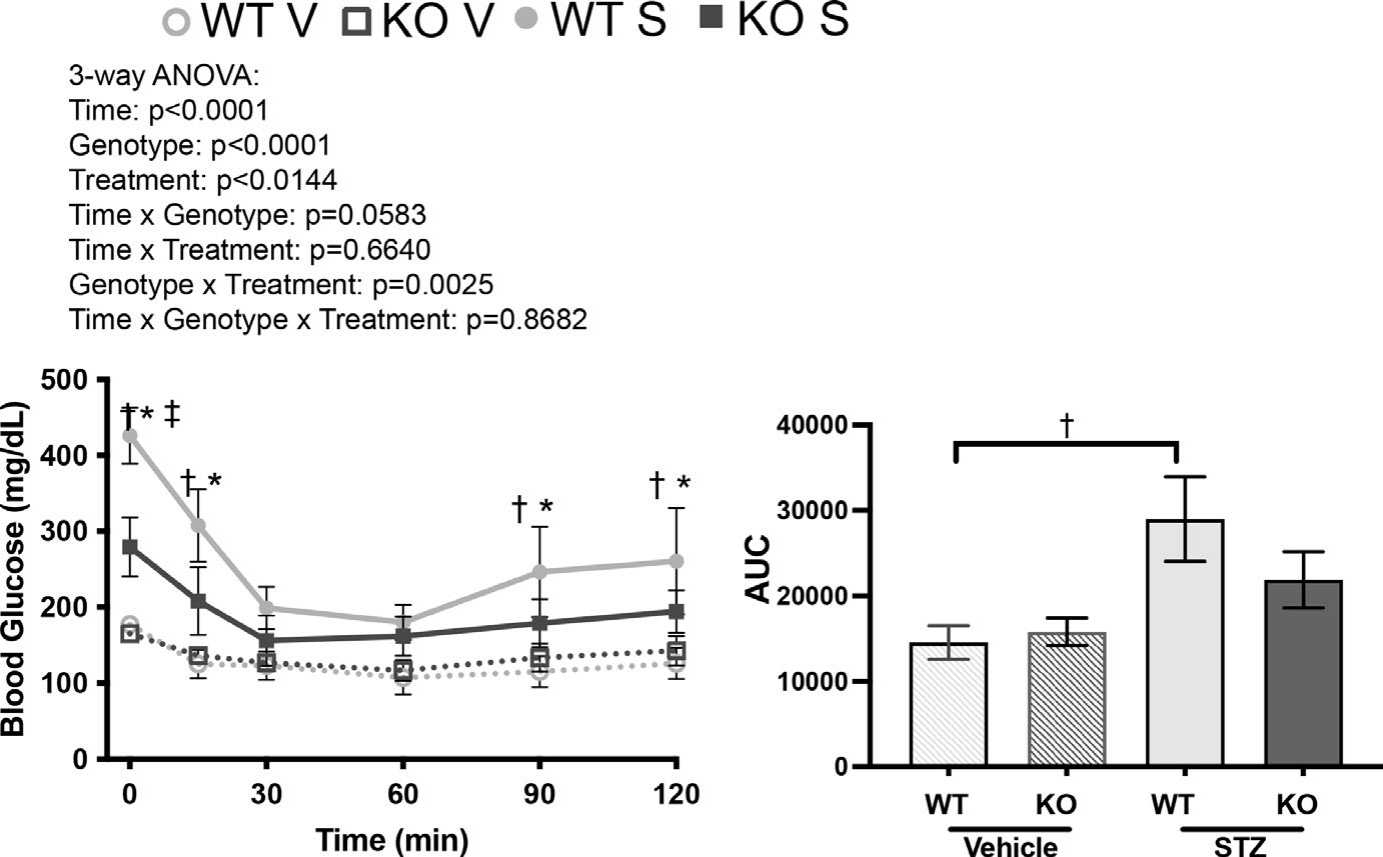
Type 1 diabetic mice remain insulin tolerant. 5 weeks after STZ challenge, male mice were subjected to an insulin tolerance test (ITT). While there was a notable difference in starting blood glucose values (2 h fasting) between diabetic Olfr1393 WT and KO, both cohorts of diabetic animals remained tolerant to insulin as noted by their decreased blood glucose values following insulin administration. Diabetic Olfr1393 KO mice were able to maintain the lower blood glucose values for up to 2 hours while the diabetic WT mice more readily increased their blood glucose. (Three‐way ANOVA followed by Tukey's post‐hoc test: *p < 0.05 WT STZ vs KO STZ; †*p* < 0.05 WT vehicle vs WT STZ; ‡*p* < 0.05 KO vehicle vs KO STZ). The area under the curve is plotted to the right with significance defined as *p < 0.05 WT STZ vs KO STZ; †*p* < 0.05 WT vehicle vs WT STZ by one‐way ANOVA followed by Tukey's post‐hoc test. (vehicle: 6 WT, 7 KO; STZ: 7 WT, 6 KO)

As a model of T1D, we anticipated that the mice would remain insulin tolerant if presented with exogenous insulin. Indeed, both WT and KO diabetic mice had a linear decline in blood glucose values from 0 to 30 min following insulin administration 5 weeks after STZ challenge (Figure 4). KO mice did have a significant reduction in the AUC and were better able to maintain normal blood glucose levels up to 120 min following insulin challenge. This tracks with their attenuated hyperglycemia and improved glucose tolerance (Figures 1 and 3).

**FIGURE 5:**
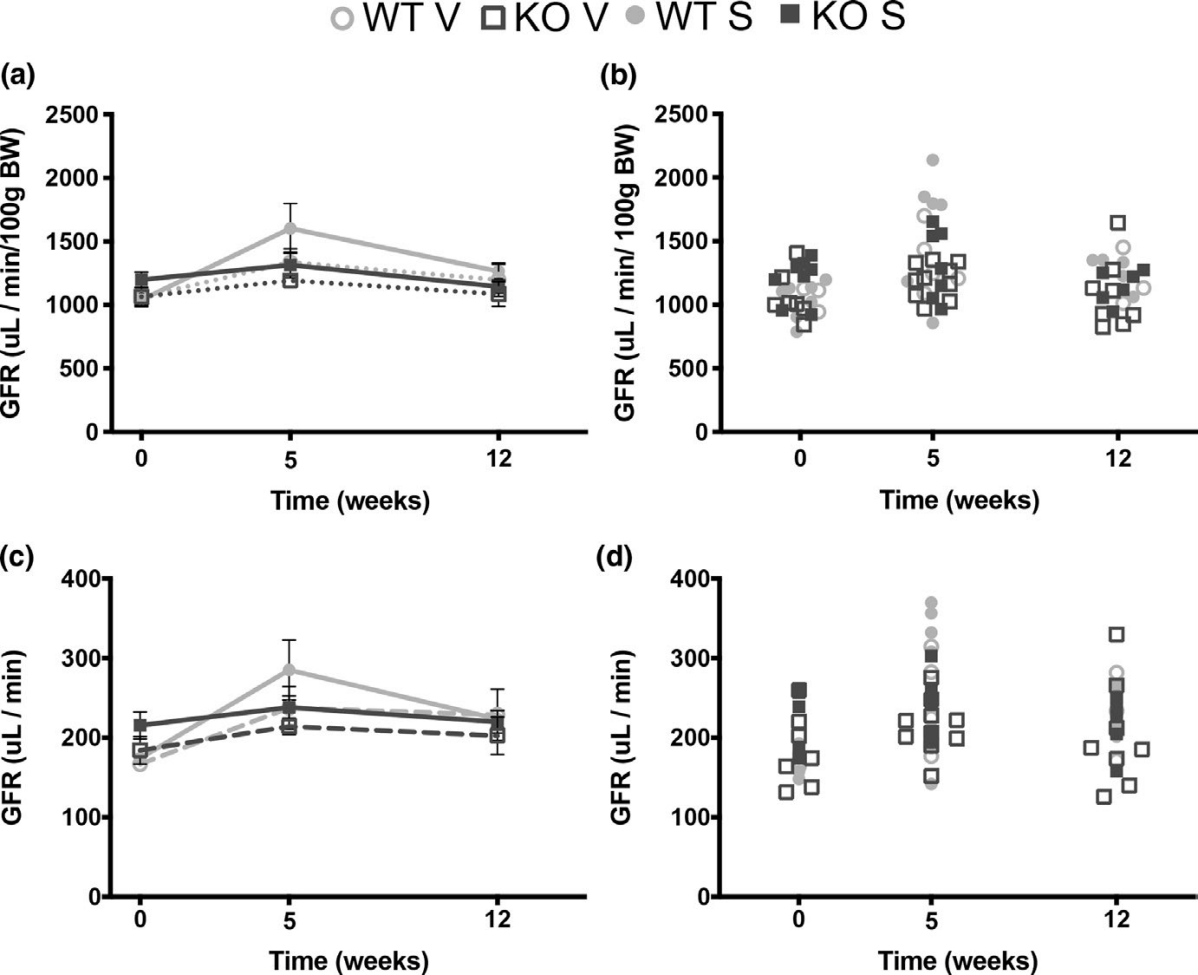
Type 1 diabetic WT and KO mice do not present with glomerular hyperfiltration. Glomerular filtration rate (GFR) was measured in Olfr1393 WT and KO mice at 0, 5, and 12 weeks following challenge with STZ (S) or vehicle control (V). Contrary to the literature, diabetic WT mice did not display hyperfiltration when GFR was calculated either as μl/min/100 g BW (a) or in μl/min (c). As seen to in the individual measurements plotted on the righthand side (b and d), at 5 weeks, GFR in WT STZ mice did trend higher than the diabetic KOs and vehicle controls, but this did not reach significance. (*N* at week 0: WT vehicle = 3, KO vehicle = 7, WT STZ = 6, KO STZ = 8; *N* at week 5: WT vehicle = 5, KO vehicle = 10, WT STZ = 6, KO STZ = 7; *N* at week 12: WT vehicle = 3, KO vehicle = 8, WT STZ = 5, KO STZ = 6)

Diabetic patients often present with glomerular hyperfiltration which has been linked to the progression of diabetic kidney disease (De Nicola et al., 2014; Mora‐Fernandez et al., 2014; Vallon & Thomson, 2012). Studies using a similar STZ treatment regimen have observed hyperfiltration by 5 weeks post‐STZ (Vallon et al., 2013) and our previous findings showed that high fat diet‐fed Olfr1393 KO mice have an attenuated hyperfiltration phenotype (Shepard et al., 2019). Thus, we anticipated that the improved glucose tolerance observed in our T1D KO mice would prevent or minimize diabetes‐induced glomerular hyperfiltration. To test this, we tracked the GFR in our animals at 0, 5, and 12 weeks post‐STZ using transdermal monitoring of FITC‐Sinistrin (Schreiber et al., 2012; Shepard et al., 2016, 2019; Figure 5). Surprisingly, we were unable to detect hyperfiltration in our diabetic animals with no statistical difference noted between our diabetic and vehicle controls at any timepoint (μl/min/100 g BW; Figure 5a). Given the change in body weight over time, we also chose to calculate the GFR for each mouse μl/min; even with this calculation there were no differences noted (Figure 5c). Finally, to determine if a subset of our mice were hyperfiltrating, we plotted the GFR of each individual mouse (Figure 5b,d). Using this analysis, at the 5‐week timepoint, it is clear that almost all of the WT diabetics have a higher GFR than the KOs and the vehicle controls. Nonetheless, this did not reach statistical significance.

**FIGURE 6:**
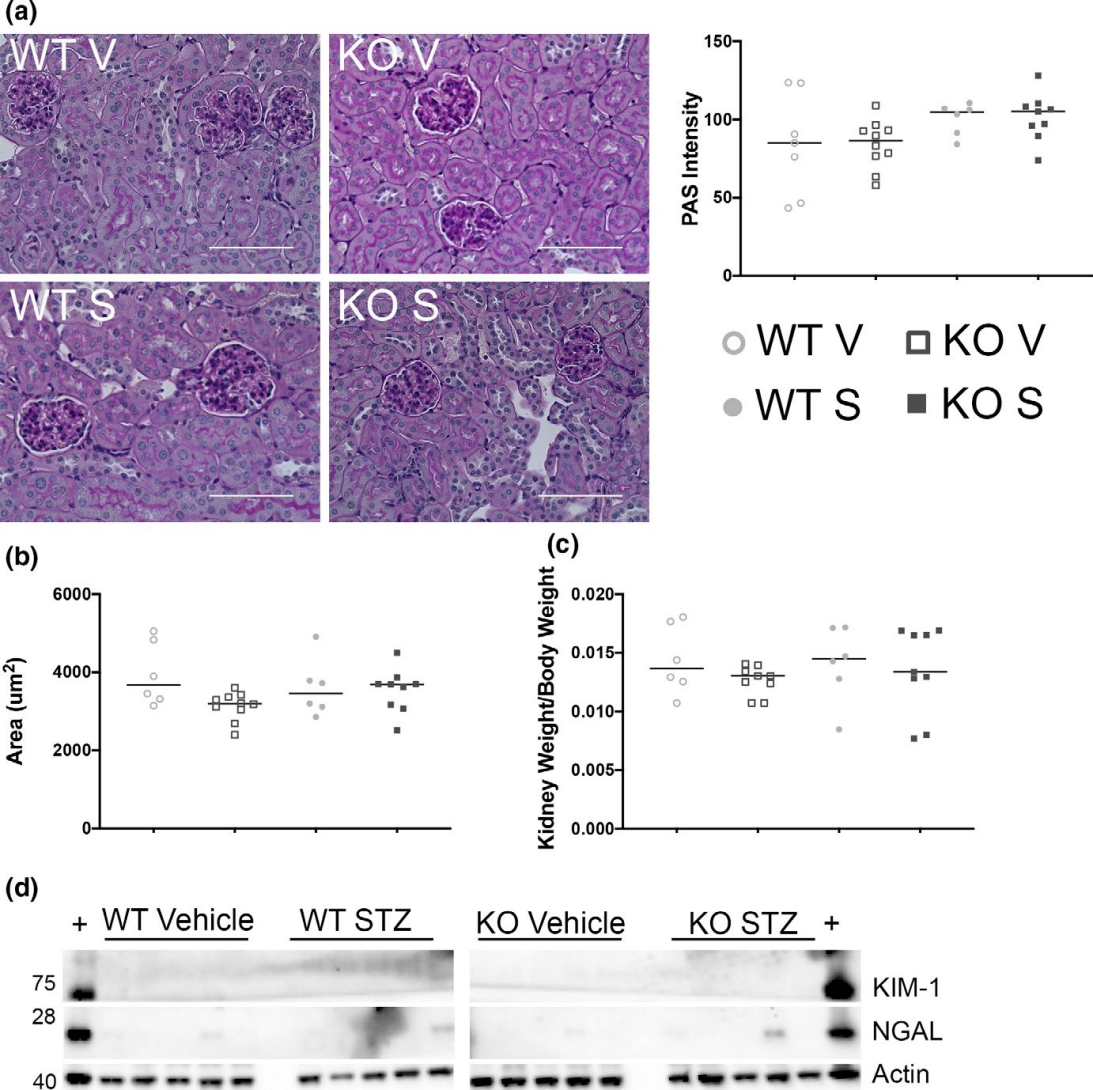
Diabetic WT and KO mice do not present with glomerular injury. 12 weeks post‐STZ challenge, kidneys were harvested from diabetic and vehicle control WT and KO male mice and stained with Periodic Acid Schiff (PAS) to detect mesangial cell expansion. PAS intensity is shown to the right (a). Total area of individual glomeruli (glomerular area + Bowman's Space) was quantified by ImageJ analysis (b). There was also no difference detected in the ratio of Kidney Weight to Body Weight in the same mice (c). Significant expression of KIM‐1 or NGAL were not detected (d). As a positive (+) control, kidney lysates from 2,8‐dihydroxyadenine were run alongside STZ and vehicle samples. In A‐C, the median value is marked by a horizontal line. (*N* for Vehicle: WT = 6, KO = 9; *N* for STZ: WT = 6, KO = 8)

In light of the lack of hyperfiltration, we evaluated whether the renal glomeruli in the diabetic mice showed signs of mesangial expansion or a change in size/number. As seen in Figure 6, there was only a slight increase in mesangial expansion noted by deep purple PAS staining within glomeruli (Figure 6a) with no corresponding change in glomerular area (Figure 6b). This correlated to similar kidney weight/body weight ratios (Figure 6c) between Olfr1393 WT and KO diabetic and controls. We also did not detect significant levels of renal KIM‐1 or NGAL, two proteins known to be upregulated upon renal damage (Figure 6d).

**FIGURE 7:**
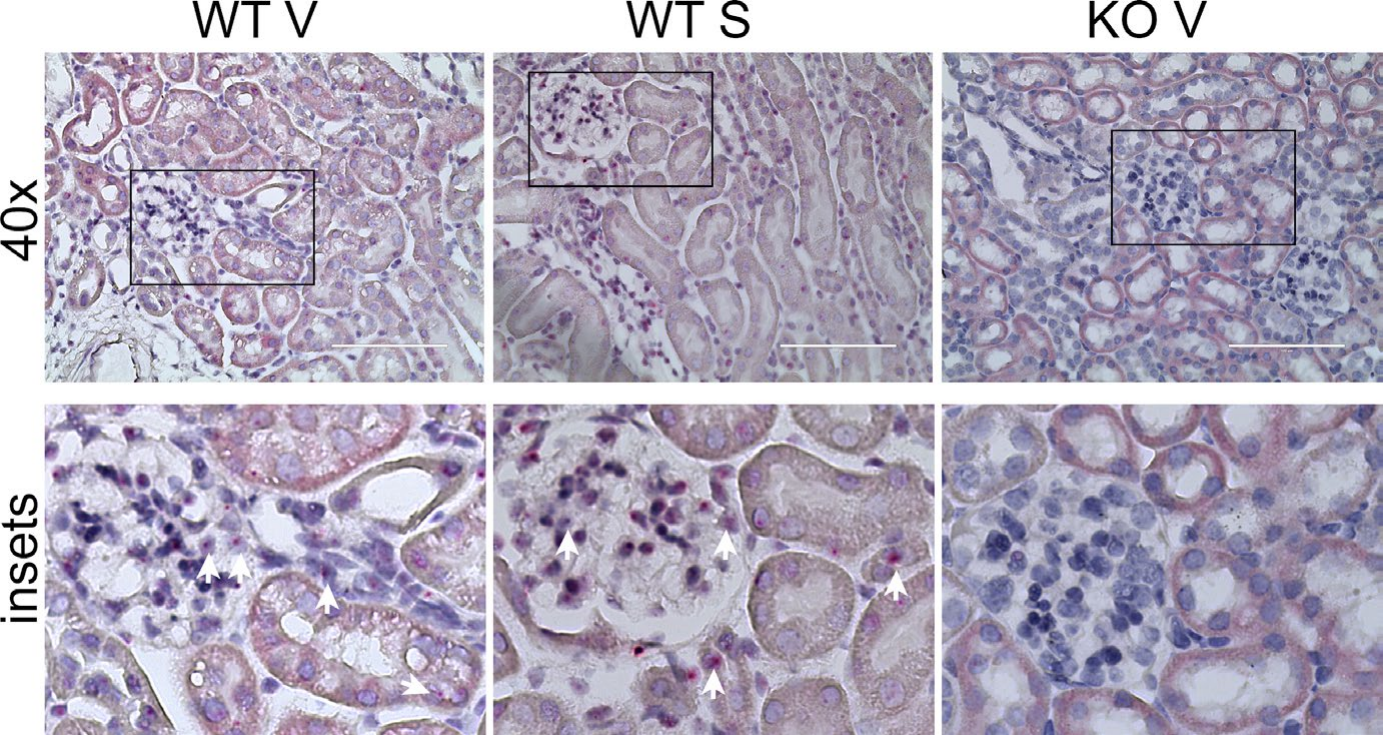
Olfr1393 is found in the renal proximal tubule and glomerulus. Paraffin sections of Olfr1393 heterozygous (+/−) and KO ( − /−) kidneys were obtained and subjected to BaseScope labeling using a custom Olfr1393 probe. Pink puncta were visible in the proximal tubule and glomeruli (see insets and arrows) of the Olfr1393 +/− kidneys, but absent in the −/− kidneys

**FIGURE 8:**
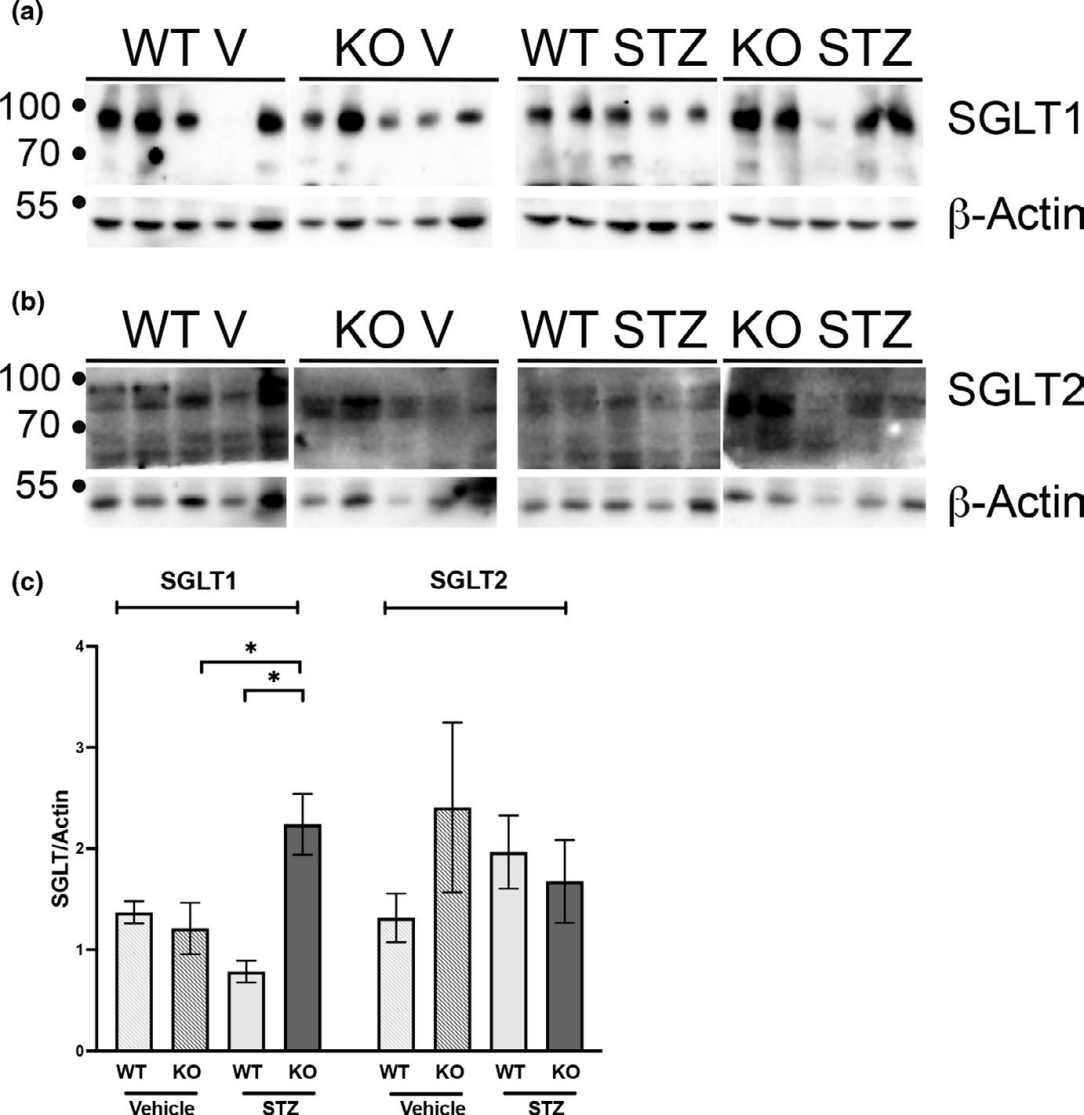
SGLT1 expression is increased in diabetic Olfr1393 KO mice. 12 weeks post‐STZ challenge, kidneys were harvested from diabetic (STZ) and vehicle control (V) male mice, lysed, and probed for SGLT1 (a) and SGLT2 (b) expression. Densitometry analysis was performed and normalized to β‐actin (c). (*N* = 5 for all samples); *p < 0.05

We previously determined that Olfr1393 is expressed in all three segments of the renal proximal tubule (via hand dissected nephron segment RT‐PCR). To confirm this, we generated a custom BaseScope probe to specifically localize this receptor. As seen in Figure 7, puncta of Olfr1393 Z probes were found in the renal proximal tubule of Olfr1393 WT kidneys but this was absent in the kidneys from the KOs. In addition to the proximal tubule, we also observed expression of Olfr1393 in the glomerulus (Figure 7) expanding the renal area where Olfr1393 signaling is known to be active. Given the localization of Olfr1393 to the renal proximal tubule, and preliminary urinary dipstick recordings suggesting increased glycosuria in the KO STZ mice (glucose dipstick: WT V – normal, KO V – normal, WT STZ – normal‐30 mg/dl, KO STZ normal‐60 mg/dl), we examined the expression of both SGLT1 and SGLT2 in kidneys from vehicle and STZ‐treated mice. As previously reported, neither SGLT1 nor SGLT2 expression is altered in healthy Olfr1393 KO (Shepard et al., 2016) mice. Consistent with other reports (Albertoni Borghese et al., 2009; Harris et al., 1986), we observed a decrease in SGLT1 in the diabetic WT mice (Figure 8). However, in diabetic Olfr1393 KO mice, SGLT1 expression was increased (Figure 8c). No changes were observed in SGLT2 expression. The slight increase in SGLT1 expression in diabetic KO mice may point to a potential mechanism for improved hyperglycemia.

## 4 DISCUSSION

### 4.1 Olfr1393 KO males are more resistant to the development of diabetes

Olfr1393 is actively involved in the process of maintaining glucose homeostasis. Under normal conditions, loss of Olfr1393 results in glycosuria, improved glucose tolerance, and a decrease in the luminal expression of SGLT1 within the proximal tubule (Shepard et al., 2016). When challenged with a high fat diet feeding, we also determined that modulating glucose handling via Olfr1393 results in an improved T2D phenotype (Shepard et al., 2019). This was accompanied by a decrease in SGLT2 expression. Finally, the data presented here suggest that Olfr1393 also has a role in T1D. Despite equivalent damage to the Islets of Langerhans following STZ treatment, Olfr1393 KO mice have a significant improvement in hyperglycemia and glucose tolerance, and can maintain lower blood glucose levels following administration of exogenous insulin. Collectively, our former and current studies implicate Olfr1393 as a modulator of hyperglycemia and glucose tolerance through a contribution to renal glucose handling.

Our current analysis of T1D was performed in mice challenged with STZ. While use of STZ gives rise to the hallmark characteristics of T1D (mainly hyperglycemia and glucose intolerance), it does so independently of the autoimmune disorder that this disease is characterized by. This antimicrobial glucose analog selectively accumulates in pancreatic β cells via the glucose transporter, GLUT2, and causes necrosis by alkylating DNA. Due to this method, off target effects of STZ have been reported, especially in those tissues that express GLUT2 including the kidney and liver (Lenzen, 2008). Additionally, as an antimicrobial agent, STZ may also give rise to changes in the gut microbiome that are independent of the reported changes that are observed in patients with diabetes (Yuan et al., 2018). We cannot rule out the possibility that STZ is producing off‐target effects in the kidney. While Olfr1393 is linked to glucose homeostasis, it is unknown if loss of this receptor leads to changes GLUT2 activity. Therefore, while Olfr1393 WT and KO mice appeared to be equally sensitive to STZ’s alkylating effects within the pancreas, we cannot rule out that there may be differential effects on the kidney. Thus, for these reasons, it may be beneficial to confirm our findings with Olfr1393 in a more physiologically relevant model such as the Akita mice who harbor a mutation in the insulin 2 gene and model monogenic diabetes, or the NOD mouse that models autoimmune‐initiated T1D.

### 4.2 Olfr1393: sex differences

Our previous characterization of Olfr1393 KO mice revealed some key sex differences; on a normal chow diet, while both males and females presented with glycosuria, this was more pronounced in the females. In addition, on a high fat diet, female KOs presented with an attenuated hyperfiltration despite an overt glucose intolerance. Collectively, this data indicates that Olfr1393 might have a greater role in female mice (Shepard et al., 2016, 2019).

Unfortunately, our efforts to characterize T1D female mice was hampered by our inability to induce diabetes in this sex. This is commonly observed in female C57BL6 mice, and extends to other, more physiologically relevant diabetic models as well (Irsik et al., 2018; Le May et al., 2006; Ostenson et al., 1989; Rossini et al., 1978; Shepard, 2019). Thus far, this resistance has been attributed to differences in sex hormones; when estrogen receptor signaling is altered (as in the case of estrogen receptor and aromatase KOs), female mice become susceptible to STZ‐induced diabetes (Le May et al., 2006). Given the importance of Olfr1393 in the female, future studies will be necessary to resolve Olfr1393’s contribution to the progression of diabetes in this sex.

### 4.3 Diabetic mice do not present with glomerular hyperfiltration

We previously observed that WT mice developed glomerular hyperfiltration following consumption of a high fat diet and this was attenuated in the Olfr1393 KOs. Thus, we hypothesized that the same trend would be present following the induction of T1D. Indeed, the literature does report that STZ‐treated mice display hyperfiltration by 5 weeks post‐challenge (Vallon et al., 2013). However, we were unable to recapitulate these findings as neither the diabetic WT nor KO male mice displayed signs of hyperfiltration despite a comparably powered study when analyzed with or without body weight correction. We also did not note any correlation between the recorded GFR and their body weight/blood glucose values. Analysis of individual mice (Figure 5) indicates that a subset of our WT mice did present with a 1.5–2× increase in their GFR at 5 weeks post‐STZ as compared to their baseline values. However, these same mice did return to “normal” at 12 weeks. Given the severe hyperglycemia that our diabetic mice were presenting with, it is puzzling that they are not hyperfiltrating. While these studies were performed on C57BL6 mice which are resistant to kidney injury, they are known to develop hyperfiltration (Vallon et al., 2013). Preliminary data suggested that plasma sodium levels were unchanged between mice which could partially explain the lack of hyperfiltration. Furthermore, our GFR measurements were performed using the continuous transdermal monitoring of FITC‐Sinistrin while Vallon and colleagues measure FITC‐Sinistrin decay from blood measurements (Vallon et al., 2013). It is possible these methodological differences could account for the differing results in the WT mice. A recent report indicates that reliance on transcutaneous measurement of FITC‐Sinistrin may be unable to accurately compute GFR in obese, diabetic animals (Mullins et al., 2020). While our mice were not obese and STZ treatment did not promote changes in body weight, it is still possible that our T1D mice had vascular dysfunction that prevented the transdermal detection of hyperfiltration. Confirmation using alternative methods could benefit our analysis. It should be noted that GFR was calculated using the 3‐compartment model which was recently found to be the optimal technique for GFR measurements (Ullah et al., 2020). However, in this study using STZ‐treated rats, hyperfiltration peaked much earlier (only 2‐weeks post‐STZ). Therefore, we also cannot rule out the possibility that a transient hyperfiltration was displayed much earlier than our anticipated 5‐week timepoint.

### 4.4 Mechanistic insight

Work from our laboratory has shown that Olfr1393 acts as a regulator of glucose homeostasis; localized to the renal proximal tubule, previous work has found that loss of this receptor also leads to mild changes in expression and/or localization of the SGLTs. On a normal chow diet, luminal expression of SGLT1 was reduced by ~25% despite no change in total protein expression (Shepard et al., 2016) and when challenged with a high fat diet, we observed a ~30% reduction in SGLT2 (Shepard et al., 2019). Here, we note an *increase* in SGLT1 expression in the diabetic KO mice with no changes in SGLT2. Within the kidneys, the SGLTs account for all glucose reabsorption (Rieg et al., 2014), and given the localization and function of Olfr1393, combined with the previously‐observed altered expression of these transporters, it is tempting to speculate that this GPCR serves as a regulator of renal glucose reabsorption. However, both our current and previous data note subtle changes in SGLT expression which alone is likely not sufficient to explain the significant attenuation in hyperglycemia observed in this study. Indeed, loss of SGLT1 was *not* associated with a reduction in fasting blood glucose levels in STZ‐challenged mice (Powell et al., 2013) although the SGLT1 KOs did display improved glucose tolerance. While SGLT2 is confined to the kidney, SGLT1 is expressed more broadly (Mather & Pollock, 2010, 2011; Shepard & Pluznick, 2017). Its main function lies in the small intestine where it contributes to post‐prandial glucose absorption. It also has a role in renal glucose reabsorption and has both known and unknown functions in other tissues including the pancreas, liver, and brain (Vrhovac et al., 2015). It is worth noting that the Olfr1393 KO mice are global KOs, and while expression is highest within the kidney, it has been found to be expressed in other tissues including the small intestine and liver (Shepard et al., 2016). Therefore, it is possible that the improved diabetic phenotypes of the KOs may be partially explained by Olfr1393’s expression outside of the kidney. Given its link to glucose reabsorption, intestinal and hepatic expression of both SGLT1 and facilitative glucose transporters could be altered in Olfr1393 KOs. In addition, Olfr1393’s role in glucose metabolism and insulin signaling warrants further study (both of which are altered in diabetic treatments DeFronzo et al., 2017; Rines et al., 2016)). In this study, we expanded the known localization of Olfr1393 to include the glomerulus. Several GLUT transporters are expressed within the glomerulus (Mather & Pollock, 2011), and it is tempting to speculate that glomerular Olfr1393 signaling plays a role in glucose homeostasis as well. Finally, while the expression changes of the renal SGLTs are mild in the Olfr1393 KOs, we cannot rule out a change in transport activity. Efforts are currently ongoing to establish whether Olfr1393 signaling contributes to glucose transport in both the kidney and intestine.

### 4.5 Olfr1393 as therapeutic target?

Olfr1393 is one of thousands of known GPCRs. As a class of receptors, GPCRs represent the largest “druggable” gene family with more than 20% of FDA‐approved drugs targeting these receptors (Lundstrom, 2009; Wacker et al., 2017). Given Olfr1393’s contribution to glucose homeostasis, it is tempting to speculate that this receptor could be a future drug target itself. Olfr1393 is a murine OR and its human ortholog remains unclear. There are several human ORs that have been found to be in the kidney providing promise that these receptors have a similar role in the modulation of blood glucose (Kalbe et al., 2016). To confirm this, studies should be performed to determine if the ligand profile of this mouse OR matches that of the potential orthologs. Olfr1393 is known to respond to small cyclic molecules containing either ketone or alcohol groups (Shepard et al., 2016). To date, the truly physiological ligand for this receptor remains a mystery. Nonetheless, the structures of the known agonists would offer enough guidance for drug development. Thus, this GPCR has translational potential if its mechanistic model can be completely elucidated.

## CONFLICT OF INTEREST

The authors (ARS, EGC, MB, and BDS) certify that they have NO affiliations with or involvement in any organization or entity with financial interest in the subject matter or material discussed in this manuscript.

## AUTHOR CONTRIBUTIONS

ARS, EGC, and BDS designed the research; ARS, EGC, and MB performed the research; ARS, EGC, and BDS analyzed the data; ARS and BDS wrote the manuscript
